# Application of telemedicine in the COVID-19 epidemic: An analysis of Gansu Province in China

**DOI:** 10.1371/journal.pone.0249872

**Published:** 2021-08-04

**Authors:** Yan Wang, Jie Yang, Huijuan Ma, Xinchun Dong, Guangmei Xie, Songning Ye, Juan Du

**Affiliations:** 1 Department of Telemedicine Consultation Center, Gansu Provincial Hospital, Lanzhou, Gansu, China; 2 Gansu Provincial Maternity and Child-care Hospital, Lanzhou, Gansu China; 3 Gansu Provincial Hospital, Lanzhou, Gansu, China; 4 Department of Human Resources, Gansu Provincial Hospital, Lanzhou, Gansu, China; University of Auckland, NEW ZEALAND

## Abstract

This paper analyzes the application of various telemedicine services in Gansu Province, China during the COVID-19 epidemic, and summarizes the experiences with these services. In addition, the satisfaction levels of patients and doctors with the application of telemedicine in COVID-19 were investigated, the deficiencies of telemedicine in Gansu were determined, and recommendations for modification were proposed. Coronavirus Disease 2019 (COVID-19) has broken out in China, and Gansu Province in Northwest of China has not been spared. To date, there are 91 local COVID-19 cases and 42 imported cases. 109 hospitals were selected as designated hospitals during the COVID-19 outbreak, and most of them were secondary hospitals. However, it was unsatisfactory that the ability of medical services is relatively low in most of secondary hospitals and primary hospitals. Therefore, we helped the secondary hospitals cope with COVID-19 by means of remote consultation, long-distance education, telemedicine question and answer (Q&A). Our practical experience shows that telemedicine can be widely used during the COVID-19 epidemic, especially in developing countries and areas with lagging medical standards.

## Introduction

### Epidemic profile of Gansu Province

Gansu Province is situated in Northwest of China. Its terrain is long and narrow, and it is approximately 1,665 km in length from east to west. The total area of Gansu is approximately 425,000 km^2^, and it has a population of more than 26 million. Gansu is one of the developing provinces in China, and the development of the economy is unbalanced [1]. Lanzhou is the provincial capital city and the political and cultural center of Gansu Province. High-quality medical resources, such as tertiary hospitals with skillful doctors and advanced medical equipment, are mainly distributed in Lanzhou. In 2019, the population of Gansu Province was 26.474 million. There were only 19 tertiary first-class hospitals in Gansu Province, 9 of which were in Lanzhou City.

Since the COVID-19 epidemic broke out in Wuhan China at the end of December 2019, the epidemic has spread to other parts of China and has attracted worldwide attention [2–4]. The Chinese government has taken swift and effective measures to address the outbreak of the epidemic [5–8]. On January 23, 2020, 2 patients were confirmed to have COVID-19 in Gansu Province. These were the first patients who were found and confirmed to have COVID-19 in this province. As of February 27, 2020, a total of 91 confirmed cases of COVID-19 and 2 related deaths had been reported, according to the data of the Gansu Provincial Health Committee. To control the spread of the epidemic in Gansu Province, the Gansu government has taken measures regarding first-level responses to major public health emergencies. A total of 109 hospitals across the province were selected as designated hospitals for the treatment of COVID-19, and the patients who were confirmed with COVID-19 throughout the province were all isolated and treated in these hospitals. This isolated treatment method could limit the flow of patients to the greatest extend and avoid further infection caused by personnel flow. Twenty-three of the 109 designated hospitals in Gansu Province were tertiary hospitals, 6 of which were located in Lanzhou city. The other 86 hospitals were secondary hospitals. Because of their relatively lower level of medical technology and less advanced medical equipment, the issue of how to meet the needs related to the diagnosis and treatment of COVID-19 patients is a huge challenge for these secondary hospitals.

### Overview of Gansu telemedicine

Telemedicine has been in used for over half a century [9]. In recent years, communication technology and sensing techniques have become increasingly developed [10], making telemedicine more popular and important in medical services. The Gansu Telemedicine Consultation Center was established in Gansu Provincial Hospital in 2007 as the first telemedicine center in Northwest of China; with 1,510 network hospitals, it is the largest telemedicine consultation network platform in China. Among all the network hospitals, there are 23 tertiary hospitals, 351 secondary hospitals and 1,136 primary hospitals. As the top tertiary hospital within the network platform, Gansu Provincial Hospital can accept the consultation cases submitted by other tertiary hospitals and all secondary hospitals, and secondary hospitals can accept the consultation cases submitted by primary hospitals. The center provides various remote medical services, such as remote consultation, remote pathology, remote imaging, remote ECG and critical care, and telemedicine education. In the COVID-19 epidemic, telemedicine has been widely used for remote consultation and long-distance education in Gansu Province due to its convenient, flexible and noncontact characteristics. On January 21, 2020, a special telemedicine platform for COVID-19 was set up, and a 24-hour free online service was implemented. According to the concrete conditions of the epidemic, three measures were proposed by the Telemedicine Consultation Center. First, the telemedicine consultation platform was extended to the isolation wards of all designated hospitals to facilitate the doctors in the wards with submitting remote consultation cases. Second, long-distance education was carried out and the medical staff in the secondary and primary hospitals were educated about the diagnosis, treatment, nosocomial infection prevention of COVID-19, and so on. Third, a telemedicine Q&A (question and answer) platform was opened, which is different from the telemedicine consultation platform. The telemedicine Q&A platform does not aim at specific cases and does not require the doctors of secondary and primary hospitals to submit cases of patients. It is only used for general answers to COVID-19-related questions. In this paper, we will analyze the application of telemedicine in the COVID-19 epidemic in Gansu, China, and summarize the experiences and deficiencies related to applying telemedicine in emergencies.

### Characteristics of remote consultation platform and software

There were two ways of remote consultation in Gansu Province: "soft video" and " hard video". Polycom audio and video systems were used for "hard video" consultation, and ordinary computers connected to internet were used for "soft video" consultation. As a unified platform of the whole province, "Beijing ICF V5.1 System" was used to submit consultation cases. "Hard video" has the advantages of high definition and good consultation experience, but the cost of equipment is high. "Soft video" has the characteristics of simple operation, convenient installation and low price. In the epidemic, In order to facilitate the doctors of secondary hospitals to carry out remote consultation in isolation wards, the "soft video" was used for teleconsultation. At present, in the remote consultation platform of " Beijing ICF V5.1 ", Gansu Provincial Hospital was the only tertiary hospital in the province that accepted consultation cases, and it was also the only tertiary hospital that assists secondary designated hospitals to solve the problems through telemedicine in the epidemic. In order to ensure that the doctors in secondary designated hospitals could fully communicate with experts, all consultations were required to communicate face to face through video system. The consultation process was as follows. The patients’ cases were reported to the expert in detail by the doctors in the secondary hospital. The experts discussed with the competent doctors after understanding the patients’ condition. Finally, the experts issued consultation reports, which were transmitted to the competent doctors through the remote consultation platform. "Beijing ICF V5.1 System" is a system with multiple functions. Besides remote video consultation, the platform can also be used for remote imaging diagnosis and remote education. In the epidemic, long-distance education and imaging diagnosis were also completed by using this platform.

## Materials and methods

### Data collection

To understand the application of the telemedicine platform during the COVID-19 epidemic in Gansu, China, we collected and analyzed the cases of differential diagnoses and the diagnosis and treatment of COVID-19 that were submitted to Gansu Provincial Hospital through the remote consultation platform from January 21 to February 29, 2020. Long-distance education and telemedicine Q&A cases were also analyzed. Ethics committee approval was obtained from the Ethics Committee of Gansu Provincial Hospital.

As users of telemedicine in the COVID-19 epidemic, the process’s value in the epidemic is well known to both doctors and patients. To understand their levels of satisfaction with telemedicine during the epidemic, different questionnaires for doctors and patients were designed, and the participants consisted of recruited people who experienced telemedicine from January 21 to February 29, 2020.

For patients who needed remote consultation, their competent doctors (i.e., doctors at the secondary hospitals) obtained their consents to record their information in patient cases and then submitted these cases to the consultation platform to apply for expert consultation. For the minors who needed remote consultation, they obtained consent from their guardians. Those who participated in the questionnaire survey were all informed of the purpose of the survey. They filled out the questionnaires if they agreed to participate, and they did not fill out the questionnaires if they did not agree to participate. Due to the large number of participants, the oral consent procedure was adopted. The study was approved by the Ethics Committee of Gansu Provincial People’s Hospital. All participants provided informed consent.

### Analysis

Descriptive statistics were used to describe the application of telemedicine and the satisfaction of doctors and patients with telemedicine during the COVID-19 epidemic in Gansu Province. The data were collected, the totals were counted and the respective percentages were calculated. The data were assessed and passed by the Ethics Committee of Gansu Provincial People’s Hospital. The data provided does not involve personal privacy.

## Results

### Remote consultation

As shown in Table 1, there were 1043 remote consultation cases related to COVID-19 from January 21 to February 29,2020. Of these cases, 570 were from the respiratory department, which was the largest number. In addition, there were 19 cases from the pediatrics department. As shown in Table 2, the distances between the secondary hospitals that submitted the consultation cases and the Telemedicine Consultation Center ranged from less than 100 kilometers to more than 1000 kilometers. Sixty-four cases came from secondary hospitals that were 500 kilometers away. Because of the traffic inconvenience and the risk of hospital infection and other related problems, it was difficult and hazardous for patients to be transferred from secondary hospitals to provincial hospitals. Thus, the application of telemedicine during the COVID-19 epidemic helped to overcome the difficulties related to patients transfers and the limited capacity of treatment. It made it possible for these patients to receive timely and high-quality treatment from secondary hospitals. The best advantage of telemedicine is its timeliness. As shown in Table 3, during the epidemic, telemedicine evidenced a very fast speed. A total of 76.03% of the examined remote consultation cases were completed within 0.5–2 hours (the time from the application to the completion of the consultation).

**Table 1:** The department of remote consultation for COVID-19 cases (n = 1,043).

Department	cases(*n*)	%
Respiratory Department	570	54.65
Radiology Department	167	16.01
Intensive Care Unit (ICU)	142	13.61
Traditional Medicine Department	71	6.81
Emergency Department	37	3.55
Pediatrics Department	19	1.82
Pharmacy Department	8	0.77
Hospital Infection Management	5	0.48
Immunorheumatology Department	4	0.38
General Surgery Department	4	0.38
Cardiology Department	4	0.38
Stomatology Department	3	0.29
Hematology Department	3	0.29
Nephropathy Department	2	0.19
Digestive Department	2	0.19
Neurology Department	2	0.19

**Table 2:** The distance between secondary hospitals that apply for COVID-19 remote consultation and telemedicine consultation center (n = 1043).

The distance (km)	cases(n)	%
≥1000	15	1.44
1000–500	49	4.70
500–300	523	50.14
300–100	200	19.18
≤100	256	24.54

**Table 3:** The time from application for teleconsultation to completed consultation (n = 1043).

The time	cases(*n*)	%
≤0.5 h	78	7.48
0.5–2 h	793	76.03
2–6 h	146	14.00
6–12 h	26	2.49

### Long-distance education

Since COVID-19 was diagnosed in Wuhan in December 2019, the medical institutions in Gansu Province have paid close attention to the development of the epidemic. Because COVID-19 is a new infectious disease, related experience in the diagnosis, treatment and occupational protection of medics is lacking in the secondary and primary hospitals of Gansu. How can the medical personnel of these hospitals be trained in a timely and safe manner? Long-distance education has been a good choice. As shown in Table 4, during the period from January 21 to February 29, 2020, training on a total of 27 subjects related to COVID-19 were conducted through the telemedicine consultation platform. A total of 15,200 medical personnel from 666 secondary and primary hospitals in Gansu Province participated in this long-distance education.

**Table 4:** Long-distance education related to COVID-19 (N1 = 27, N2 = 666, n = 15,200).

Contents	Times (N1)	Number of hospitals (N2)	Number of participants (n)
Diagnosis and treatment of COVID-19	2	85	1,100
Differential diagnosis of COVID-19	2	46	1,200
Management of Fever Clinic	12	215	8,000
Specimen Collection of Patients with COVID-19 and Laboratory safety of Nucleic Acid Detection of specimens	1	26	1,000
Personal protection of medical staff in COVID-19 epidemic	6	98	1,300
Disinfection of Medical Environment	2	98	1,300
How to deal with Medical Waste of COVID-19 Patients	2	98	1,300

### Telemedicine Q&A

In addition to remote consultations and long-distance education, telemedicine question and answer (Q&A) platform also played an important role during the COVID-19 epidemic. As shown in Table 5, 167 questions from secondary and primary hospitals were answered through the platform from January 21 to February 29, 2020, of which 62 (37.13%) questions were related to the control of hospital infections during the COVID-19 epidemic.

**Table 5:** The issues raised on the telemedicine Q&A platform (n = 167).

Related questions	*n*	%
Related to Nursing Care for Patients with COVID-19	55	32.93
Related to Hospital infection control	62	37.13
Related to Psychological Guidance for patients with COVID-19	25	14.97
Related to Psychological Guidance for Medical staff in isolation wards	25	14.97

### Survey of the satisfaction of medical personnel and patients with telemedicine

Twenty-three provincial experts who had experienced telemedicine in the COVID-19 epidemic participated in the satisfaction survey. Twenty-three valid questionnaires were collected. As shown in Table 6, all the experts agreed that telemedicine was useful during the COVID-19 epidemic, especially in the areas of "saving time" and "avoiding hospital infections".

**Table 6:** The satisfaction survey of provincial experts who had experienced telemedicine in the COVID-19 epidemic (*n* = 23).

Items	*n*	%
**What types of telemedicine did you participate in during the COVID-19 epidemic?**		
Remote consultation	23	100.00
Long-distance education	11	47.83
Long-distance Q&A	5	21.74
**Do you think that telemedicine was useful during the COVID-19 epidemic?**		
Useless	0	0.00
Useful	23	100.00
Uncertain	0	0.00
**What were the benefits of using telemedicine in COVID-19 epidemic?**		
Saved time	23	100.00
Effective use of provincial expert resources	20	86.96
Avoided hospital infection	22	95.65

Sixty-one doctors in secondary hospitals who experienced telemedicine in the COVID-19 epidemic participated in the satisfaction survey, and 61 valid questionnaires were collected. As shown in Table 7, 91.80% of the surveyed doctors were satisfied the results of telemedicine during the COVID-19 epidemic. All the participants thought that telemedicine was a good choice to aid in fighting the epidemic.

**Table 7:** The satisfaction survey of doctors in secondary hospitals who had experienced telemedicine in the COVID-19 epidemic (*n* = 61).

Items	*n*	%
**What types of telemedicine did you participate in during the COVID-19 epidemic?**		
Tele-consultation	61	100.00
Long-distance education	61	100.00
Long-distance Q&A	7	11.48
**Are you satisfied with the results of telemedicine during COVID-19?**		
Unsatisfied	0	0.00
Neutral	5	8.20
Satisfied	56	91.80
**Do you think the feedback for the consultation results is timely?**		
Not timely	0	0.00
Timely	61	100.00
**Are you satisfied with the professionalism of the provincial experts in providing teleconsultation?**		
Unsatisfied	0	0.00
Neutral	2	3.28
Satisfied	59	96.72
**Do you think telemedicine is a good choice for dealing with the epidemic?**		
No	0	0.00
Yes	61	100.00
Uncertain	0	0.00
**Does telemedicine contribute to solving the problem of the unbalanced distribution of medical resources and inadequate diagnosis/treatment capacity of COVID-19 in the designated hospitals?**		
No	0	0.00
Yes	58	95.08
Uncertain	3	4.92
**Are you willing to use telemedicine to solve problems in diagnosis and treatment in the future?**		
No	0	0.00
Yes	61	100.00
Uncertain	0	0.00

As shown in Table 8, 81 patients who experienced teleconsultation during the COVID-19 epidemic participated in the satisfaction survey, and 76 valid questionnaires were collected. A total of 68.42% of the patients were satisfied with the results of remote consultation in the COVID-19 epidemic, and 92.11% of the patients were willing to introduce remote consultation to their family and friends.

**Table 8:** The survey of satisfaction of patients who had experienced teleconsultation in the COVID-19 epidemic (*n* = 76).

Items	*n*	%
**Have you ever heard of teleconsultation before?**		
No	39	51.32
Yes	37	48.68
**Have you ever used teleconsultation before?**		
No	70	92.11
Yes	6	7.89
**Does this remote consultation have your consent?**		
No	0	0.00
Yes	76	100.00
**Was the telemedicine experience convenient?**		
No	0	0.00
Yes	70	92.11
Uncertain	6	7.89
**Are you satisfied with the results of teleconsultation in the COVID-19 epidemic?**		
Unsatisfied	0	0.00
Neutral	24	31.58
Satisfied	52	68.42
**Do you think it is necessary to use the teleconsultation during the COVID-19 epidemic?**		
No	2	2.63
Yes	71	93.42
Uncertain	3	3.95
**Would you like to introduce teleconsultation to your family or friends?**		
No	0	0.00
Yes	70	92.11
Uncertain	6	7.89

## Discussion

COVID-19 has become a global emergency. According to The World Health Organization, as of March 13, 2020, more than 132,000 cases of COVID-19 have now been reported from 123 countries and territories [11]. Among these countries are developing countries, such as Iran, Thailand, Saudi Arabia, and Pakistan, in addition to developed countries. Cooperation between countries is necessary, and mutual assistance between different regions is also needed. Many methods can be adopted to encourage this cooperation and assistance, and telemedicine is one of the most effective methods, especially in developing countries and areas. Telemedicine can be achieved because IT (information technology) has been rapidly spreading globally [12]. This achievement was confirmed by Gansu Province of China during the COVID-19 epidemic.

Medical development is unbalanced in Northwest China’s Gansu Province [13]. Gansu Province has 14 cities and 86 counties. The population is 25.57 million. There are a total of 718 medical institutions in the province, including 19 tertiary first-class hospitals, of which 9 are in Lanzhou, the provincial capital. The medical service ability is low in most secondary and primary hospitals. The Telemedicine Consultation Center was established in Gansu Provincial Hospital in 2007. It can provide remote consultation, remote imaging diagnosis, remote pathological diagnosis, remote training and other services. The center was established with the support of Gansu provincial government and Gansu provincial health administrative department. It is the only telemedicine consultation center in the province. Gansu Provincial Hospital is responsible for the daily management and operation of the center. The establishment of consultation center has played an important role in solving the imbalance of medical resources distribution in the whole province. It has also promoted medical reform in Gansu Province. By using telemedicine, patients in local areas were well treated, the risks of disease spread that occurs during the transfer process was avoided, and medical expenses were saved [14]. In addition, the abilities of doctors in secondary and primary hospitals were improved by their constantly communication with provincial experts constantly.

Over the past 13 years, there are 52 medical departments involved in remote consultation.The Gansu Telemedicine Consultation Center has completed more than 60,000 cases, dealt with 43 emergency events, and accumulated much experience. During the COVID-19 epidemic, the center’s experience was further enriched by lessons learned from their newly implemented measures. First, to facilitate the special needs of remote consultation during an epidemic, the consultation platform should be flexible and mobile, with the possibility of extension to different wards. In addition, the consultation platform should be convenient for medical personnel in isolation wards to operate. Second, various telemedicine services should be carried out, such as remote consultations, remote imaging and remote education. Special attention should be paid to long-distance training during epidemic of respiratory diseases. Reducing the number of personal gatherings as much as possible is needed during epidemics of respiratory diseases; thus, medical staff trainings, conference transmissions and policy releases can be carried out through a long-distance training platform, and the infections caused by humoral transmission and contact transmission can be avoided to some extent. Third, according to the needs of different emergencies, the Telemedicine Consultation Center should organize experts in different fields to form groups to provide comprehensive diagnosis and treatment opinions for difficult cases. With the help of remote consultation, all patients with COVID-19 were effectively treated and discharged. None of the medical staff were infected. Because all the guidance was given through the network, all the provincial experts who participated in the consultation avoided the risk of occupational exposure.

Based on the results of the satisfaction survey, medical staff have a high level of awareness and satisfaction of telemedicine in regard to the COVID-19 epidemic in Gansu, China. However, patients’ awareness of telemedicine is relatively lower, which is currently one of the most important deficiencies in the development of telemedicine in Gansu Province. Many patients have never used or even heard of telemedicine before. However, what is inspiring is that when they experience remote consultation, most patients are satisfied with it. In the future, it is important to expand the publicity of telemedicine and inform more patients know about it.

In other words, telemedicine can be used in emergency events, such as the COVID-19 epidemic. Its function has been tested in practice in Gansu Province. Of course, there are still related deficiencies as well. Patients’ awareness is a primary barrier that may limit the extent of telemedicine adoption. Thus, we will further expand the publicity and diversity of telemedicine services, pay attention to the value of these services in emergencies, and help more patients learn to enjoy the conveniences of telemedicine.

From March 5 to March 15, 2020, 42 new imported cases of COVID-19 were confirmed in Gansu Province. Of these cases, 37 patients were from Iran, 4 were from Saudi Arabia, and one was from Egypt. They were all treated in one of the designated hospitals. Thus, telemedicine continues to play an important role in the treatment of these COVID-19 patients.

## Supporting information

S1 File (PDF)

S2 File (PDF)

S3 File (PDF)

S1 Questionnaire (PDF)
