# Supplementary material for: Application of telemedicine in the COVID-19 epidemic: An analysis of Gansu Province in China
Source: PLoS One. 2021 Aug 4;16(8):e0249872. doi: 10.1371/journal.pone.0249872 (PMC8336882; doi:10.1371/journal.pone.0249872)
Supplement: S1 File — (PDF) [file pone.0249872.s001.pdf]

新冠肺炎期间参与过远程会诊的患者

对远程会诊的满意度调查

亲爱的患者：感谢您参与此次关于远程医疗的满意度调查！若您愿意参与此次调查并同意将您的数据进行汇总分析后公布，则请您填写该调查表；若您不同意则请拒绝填写该表。无论您是否填写调查表，您的个人信息，包括姓名、性别、年龄，均不会出现在任何公开场合。感谢您的参与！

1、您曾经听说过远程会诊吗？

☐没有听说过　☐听说过

2、您曾经使用过远程会诊吗？

☐没有使用过　☐使用过

3、此次远程会诊是否争取到您的同意？

☐是　☐没有

4、你是否认为远程会诊方便实用吗？

☐是　☐否　☐不确定

5、您对新冠肺炎期间远程会诊的结果满意吗？

☐不满意　☐一般　☐满意

6、您认为新冠肺炎期间使用远程会诊是否必要？

☐不必要　☐必要　☐不确定

7、您愿意将远程会诊介绍给您的家人或朋友吗？

☐不愿意　☐愿意　☐不确定
