# Supplementary material for: Application of telemedicine in the COVID-19 epidemic: An analysis of Gansu Province in China
Source: PLoS One. 2021 Aug 4;16(8):e0249872. doi: 10.1371/journal.pone.0249872 (PMC8336882; doi:10.1371/journal.pone.0249872)
Supplement: S2 File — (PDF) [file pone.0249872.s002.pdf]

The survey of satisfaction of patients who had experienced tele-consultation in the COVID-19 epidemic

Dear patient: Thank you for your participation in the survey! If you would like to participate in the survey and agree to publish your data after summary analysis, please fill in this questionnaire. If you disagree, please refuse to complete this questionnaire. Your personal information, including your name, gender and age, will not appear in any public place, whether you fill out the questionnaire or not. Thank you for your participation!

1、Have you ever heard of tele-consultation before?

□No □Yes

2、Have you ever used tele-consultation before?

□No □Yes

3、Does this remote consultation have your consent?

□No □Yes

4、Was the telemedicine convenient?

□No □Yes □Uncertain

5、Are you satisfied with the results of tele-consultation in the COVID-19 epidemic ?

□Unsatisfied □Neutral □Satisfied

6、Do you think it is necessary to use the tele-consultation in the COVID-19 epidemic?

□No □Yes □Uncertain

7、Would you like to introduce tele-consultation to your family or friends?

□No □Yes □Uncertain
