# Supplementary material for: Application of telemedicine in the COVID-19 epidemic: An analysis of Gansu Province in China
Source: PLoS One. 2021 Aug 4;16(8):e0249872. doi: 10.1371/journal.pone.0249872 (PMC8336882; doi:10.1371/journal.pone.0249872)
Supplement: S3 File — (PDF) [file pone.0249872.s003.pdf]

Questionnaire on telemedicine satisfaction of medical personnel during covid-19

Dear doctor: Thank you for your participation in the survey of medical staff's satisfaction with telemedicine during COVID-19! If you would like to participate in the survey and agree to publish your data after summary analysis, please fill in this questionnaire. If you disagree, please refuse to complete this questionnaire. Your personal information, including your name, gender and age, will not appear in any public place, whether you fill out the questionnaire or not. Thank you for your participation!

1. Gender: □male □female
2. Age: ________years old
3. Occupation: □doctor □nurse
4. Professional title:
   □chief physician □associate chief physician □attending physician
   □chief nurse □deputy chief nurse □nurse-in- charge

If you are a provincial expert, please fill in the following survey contents:

1. What types of telemedicine did you participate in the COVID-19 epidemic?
   □Remote consultation □Long-distance education
   □Long-distance Q&A
2. Do you think the telemedicine is useful in the COVID-19 epidemic?
   □Useless □Useful □Uncertain
3. What were the benefits of using telemedicine in COVID-19 epidemic?
   □Save time□ Effective use of provincial expert resources □Avoid hospital infection □other__

If you are a doctor in secondary of primary hospital, please complete the following survey:

1.What types of telemedicine did you participate in the COVID-19 epidemic?
   □Remote consultation □Long-distance education
   □Long-distance Q&A
2. Do you think telemedicine is a good choice to deal with the epidemic?
   □No □Yes □Uncertain
3.Is telemedicine contribute to solve the problem of unbalanced distribution of medical resources and inadequate diagnosis/ treatment capacity of COVID-19 in the designated hospitals?
   □No □Yes □Uncertain
4.If you have attended remote consultation during COVID-19, are you satisfied with the results of the consultation
   □Unsatisfied □Neutral □Satisfied
5. Do you think the feedback of consultation results is timely?
   □Not timely □Timely
6. Are you satisfied with the professionalism of the provincial experts providing consultation?
   □Unsatisfied □Neutral □Satisfied
7. Are you willing to use telemedicine to solve the problems in diagnosis and treatment in the future?
   □No □Yes □Uncertain
