# Supplementary material for: Application of telemedicine in the COVID-19 epidemic: An analysis of Gansu Province in China
Source: PLoS One. 2021 Aug 4;16(8):e0249872. doi: 10.1371/journal.pone.0249872 (PMC8336882; doi:10.1371/journal.pone.0249872)
Supplement: S1 Questionnaire — (PDF) [file pone.0249872.s004.pdf]

新冠肺炎期间医务人员对远程医疗满意度调查表

亲爱医生：感谢您参与此次新冠肺炎医务人员对远程医疗的满意度调查！若您愿意参与此次调查并同意将您的数据进行汇总分析后公布，则请您填写该调查表；若您不同意则请拒绝填写该表。无论您是否填写调查表，您的个人信息，包括姓名、性别、年龄，均不会出现在任何公开场合。感谢您的参与！

1、性别：☐男　☐女
2、年龄：________岁
3、职业：☐医生　☐护师
4、职称：☐主任医师　☐副主任医师　☐主治医师
☐主任护师　☐副主任护师　☐主管护师
5、专业：________

若您是省级专家，请您填写以下调查内容：

1、在新冠肺炎期间，您参与过哪种形式的远程医疗：
☐远程会诊　☐远程培训　☐远程问答　☐其他
2、您认为在新冠肺炎流行期间开展远程医疗是否有用：
☐没有用　☐有用　☐不确定
3、若您认为新冠肺炎流行期间开展远程医疗有用，那么有哪些用处：
☐节约时间　☐有效利用省级专家资源　☐避免医院感染　☐其他

若您是基层医生，请您填写以下调查内容：

1、在新冠肺炎期间，您参与过哪种形式的远程医疗：
☐远程会诊　☐远程培训　☐远程问答　☐其他
2、您认为远程医疗是应对新冠肺炎疫情的一个好的选择吗：
☐不是　☐是　☐不确定
3、您认为远程医疗是否有助于解决新冠肺炎期间定点治疗医院医疗资源分布不均及诊疗能力不足的问题：
☐不能　☐能　☐不确定
4、若新冠肺炎期间您参加了远程会诊，您对会诊结果是否满意：
☐不满意　☐一般　☐满意
5、您认为远程会诊结束后，会诊结果回复是否及时：
☐不及时　☐及时
6、您对提供会诊的省级专家的专业性是否满意：
☐不满意　☐一般　☐满意
7、今后您是否愿意继续使用远程医疗解决您所遇到的疑难问题：
☐不愿意　☐愿意　☐不确定
